# Twitter misogyny associated with Hillary Clinton increased throughout the 2016 U.S. election campaign

**DOI:** 10.1038/s41598-023-31620-w

**Published:** 2023-03-31

**Authors:** Morgan Weaving, Thayer Alshaabi, Michael V. Arnold, Khandis Blake, Christopher M. Danforth, Peter S. Dodds, Nick Haslam, Cordelia Fine

**Affiliations:** 1 School of Historical and Philosophical Studies, The University of Melbourne, Parkville, VIC Australia; 2 Melbourne School of Psychological Sciences, The University of Melbourne, Parkville, VIC Australia; 3 Advanced Bioimaging Center, UC Berkeley, Berkeley, CA USA; 4 Computational Story Lab, Vermont Complex Systems Center, MassMutual Center of Excellence for Complex Systems and Data Science, University of Vermont, Burlington, VT USA

**Keywords:** Psychology, Human behaviour

## Abstract

Online misogyny has become a fixture in female politicians’ lives. Backlash theory suggests that it may represent a threat response prompted by female politicians’ counterstereotypical, power-seeking behaviors. We investigated this hypothesis by analyzing Twitter references to Hillary Clinton before, during, and after her presidential campaign. We collected a corpus of over 9 million tweets from 2014 to 2018 that referred to Hillary Clinton, and employed an interrupted time series analysis on the relative frequency of misogynistic language within the corpus. Prior to 2015, the level of misogyny associated with Clinton decreased over time, but this trend reversed when she announced her presidential campaign. During the campaign, misogyny steadily increased and only plateaued after the election, when the threat of her electoral success had subsided. These findings are consistent with the notion that online misogyny towards female political nominees is a form of backlash prompted by their ambition for power in the political arena.

## Introduction

As the first female presidential nominee for the United States, Hillary Clinton experienced a deluge of misogyny. Throughout her campaign, public slogans about Clinton touted, “Life’s a bitch: don’t vote for one,” and, “Hillary sucks, but not like Monica”^1^. This misogyny was especially apparent online, where Clinton received sexist comments so frequently that her team had, “no idea how to deal with it”, according to a former aide^2^.

The online harassment experienced by Clinton is not a unique phenomenon. A survey of 235 Australian councillors found that 49% of women reported receiving offensive online content during their term, as opposed to 35% of men. More than a third of the women (38%, compared to 10% of men) reporting received derogatory remarks focusing on their gender specifically^3^. In the UK, a 2017 BBC survey of 73 female MPs revealed that nine out of ten reported receiving online abuse, and a third had considered quitting as a result^4^. Online misogyny towards female politicians has become a widespread and troubling phenomenon that threatens female political participation.

Given female political participation is a key component of resolving gender inequities worldwide^5^, it is important to know what triggers online misogyny towards female politicians. Backlash theory suggests that female leaders face negative social consequences because they violate gender stereotypes that proscribe power-seeking in women^6^. Prior research on backlash has focused on the penalties female leaders face in their perceived hireability and likeability^7,8^. However, backlash may also underlie the online misogyny that targets female leaders.

## Backlash theory

Backlash theory begins with the assumption that gender stereotypes are not only descriptive, but also prescribe how males and females *should* act^9^. Research examining the prescriptive element of gender stereotypes has found that women are expected to act communally and avoid dominance, whilst men are expected to be agentic and independent and avoid weakness, emotionality, or shyness^10^.

Building on this research, backlash theory argues that prescriptive stereotypes act as social norms, violations of which cause negative reactions because they threaten the social status quo. Studies have repeatedly found that women who violate prescriptive stereotypes by acting dominantly are disliked more than men and are viewed as less hirable, even though they are seen as similarly competent to men^6^. Rudman et al.^8^ argue these penalties work to maintain the existing gender hierarchy, as dominant behaviors are seen as high in status, and therefore proscribing these behaviors among women maintains their relatively lower status. Examining the motives behind these penalties, research has found that backlash against women is particularly strong among people who are dispositionally inclined (or experimentally induced) to maintain the gender status quo of male dominance^8^. These findings suggest that the social penalties directed at dominant women are driven, in part, by the desire to defend the gender hierarchy.

Applying backlash theory to the political arena, experimental research has found that the expression of power-seeking intentions by political candidates negatively impacts voting preferences for female, but not male, politicians^11^. Outside of the laboratory, research has found that women who were more dominant on the Turkish legislative floor were significantly less likely to get renominated and promoted in their party ranks, whilst the reverse was true for men^12^. Other research has revealed that in the first month of the 2020 U.S. democratic presidential primaries, female (vs. male) presidential contenders received less positive ratings on warmth and likability, though this effect diminished over time^13^.

## Misogyny and backlash

Research on backlash has predominantly focused on the penalties dominant women face in their perceived likeability, hireability or upward mobility. Little research has examined whether backlash underlies misogyny. Yet, recent theorizing argues that gendered hostility—including gendered slurs, sexual objectification, and threats of sexual violence—is used to police female gender norms^14^. Supporting this, research has found that gendered harassment is typically directed towards women who violate feminine ideals, suggesting that it is motivated by a desire to punish gender deviants^15^. In online spaces, qualitative research also suggests that ‘gendered trolling’ typically targets women who defy gender norms by firmly asserting their opinions online^16^.

Social media sites have erupted in popularity in the past decade, and are an increasingly important venue for political expression^17^. This growth makes platforms like Twitter an important forum for studying social dynamics in politics, and their accessible data a powerful source of naturalistic social interactions. Indeed, a growing number of researchers have extracted valuable psychological insights from such data in recent years^18–20^. Continuing with this trend, the current study collated and analyzed Twitter data to examine the processes that underlie the online misogyny experienced by female politicians.

To explore the possibility that online misogyny towards female politicians is a form of backlash, we examined the trajectory of misogynistic tweets in a real-world historical context: the campaign of the first U.S. female presidential candidate and its aftermath. Specifically, we investigated whether misogyny towards Hillary Clinton increased after she engaged in power-seeking by announcing her presidential campaign. We therefore predicted that (a) the relative frequency of misogynistic tweets would show a rising trend after her announcement, and (b) this trend would depart in an upward direction from the pre-announcement trend.

In line with prior research, we assume that backlash is dependent on the visibility of role-violations, as it is the expressive nature of these violations that threatens social hierarchies, and therefore elicits hostile reactions^6,21^. Providing evidence of this association, research examining uncivil Twitter messages towards politicians has uncovered a significant interaction between Twitter visibility and gender, which showed that women received more uncivil messages as their follower count increased, but this effect was significantly weaker for men^22^. With this research in mind, we examined whether misogynistic reactions to Clinton were greater when she received more attention, as her role incongruity would have been more visible, and may have induced greater threat. Specifically, we investigated whether there was an association between the frequency of references to Hillary Clinton on Twitter and the relative frequency of misogynistic language associated with Clinton on Twitter. By examining the *relative* frequency of misogynistic language, we explored whether this association exists, over and above what would be expected due to the increase in references to Clinton alone.

Finally, we investigated whether there was a change in the relative frequency of misogynistic language after the election. Theoretically, it is plausible that the frequency of misogynistic language towards Clinton would decrease after her electoral defeat, as the ‘threat’ of Clinton’s win was no longer present. However, it is also feasible that Donald Trump’s win would act as a license for greater hostility towards Clinton, due to his proliferation of misogynistic rhetoric^23–25^. Because of these diverging theories, we made no specific hypothesis about how Clinton’s electoral defeat would influence misogynistic language.

## Methods

We used two complementary analytic strategies to examine our hypotheses. One strategy compared the relative frequency of misogynistic language used in tweets mentioning Hillary Clinton across three distinct periods: before Clinton announced her candidacy, during the election campaign, and after the election. The second strategy examined the relationship between the relative frequency of misogynistic language directed towards Clinton and the attention towards her on Twitter across the entire study period. To measure that attention, we identified the frequency of Hillary Clinton mentions on Twitter.

### Clinton mentions on Twitter

We used the Storywrangler API to find mentions of Clinton using the following terms (“Hillary”, “HillaryClinton”, “@HillaryClinton”) between 2014 and 2018^26^. We used ‘Hillary’ to increase the number of tweets used in the study. We are confident this search did not inappropriately identify tweets to unrelated individuals, as there are no other public-facing Hillary’s whose names are spelt identically, and hashtag searches show that all commonly collocating terms are related to Clinton (e.g., trump, politics, liberal). Storywrangler uses a random 10% sample of all English-language public Tweets, collected via Twitter’s Decahose API, to measure how often words and phrases (also known as n-grams) are used on Twitter. For each term, Storywrangler extracted the number of times the term occurred each day.

### Misogynistic language in Clinton tweets

**Figure 1:**
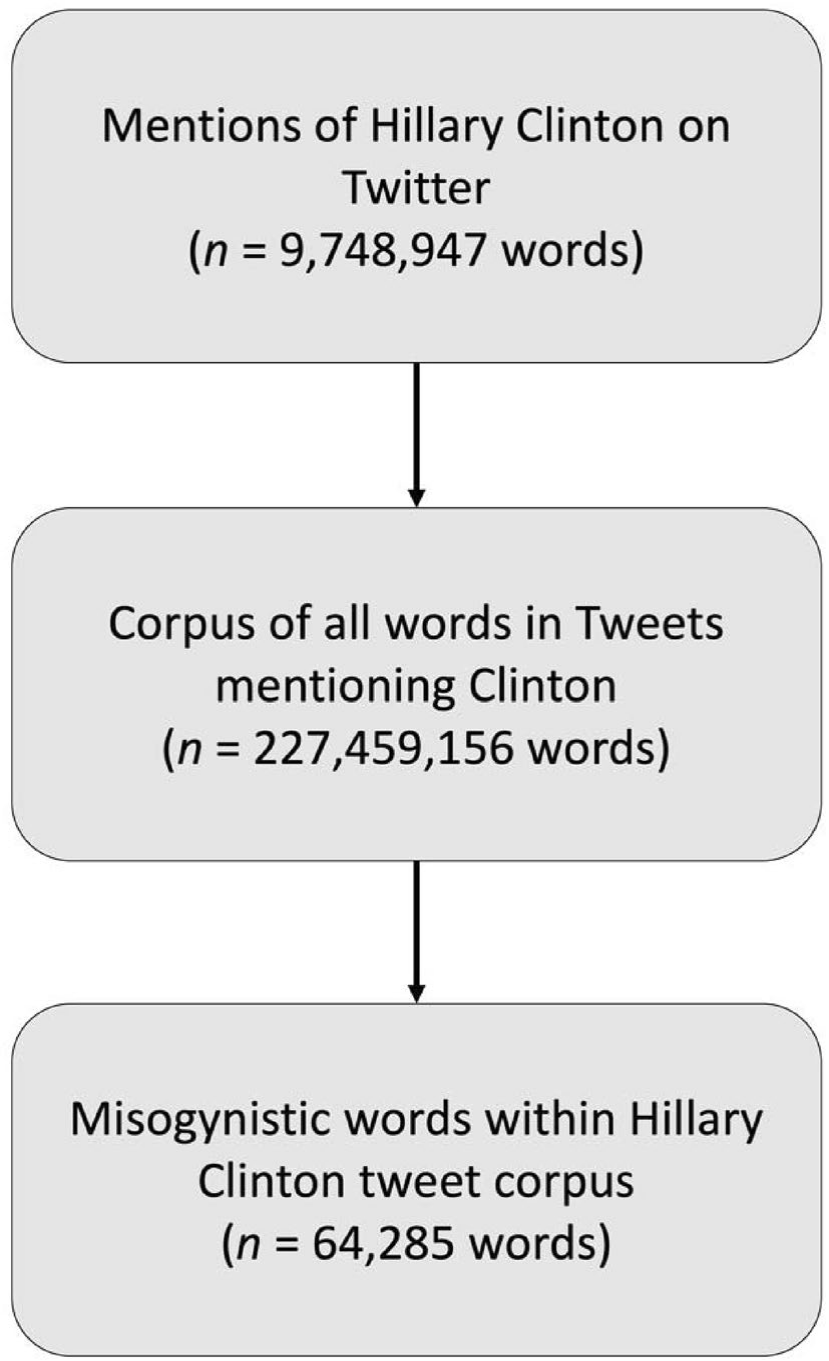
Data extraction flowchart.

Using the three Hillary Clinton search terms as anchor n-grams, we created a topic-based corpus of tweets to track and investigate linguistic patterns in Hillary Clinton tweets (see Fig. 1)^26^. Within the Hillary Clinton tweet corpus, we counted the number of misogynistic n-grams using search terms derived from Blake et al.^18^ (e.g. bitch, whore, tramp; generated from hashtag finders and manual searches of misogynistic language on Twitter). Blake et al. found that these search terms, when combined with nouns referring to women, were unambiguously misogynistic in 92.7% of 1000 tweets checked by manual coders^18^. We subsequently calculated the relative frequencies of the searched words by normalizing the count of misogynistic words by the total frequency of all words within the Hillary Clinton corpus for each day. The relative frequency of Twitter misogyny therefore refers to the proportion of all words in the Hillary Clinton corpus that were misogynistic, according to our dictionary, for each day.

### Interrupted time series

Our corpus contained 9,748,947 mentions of Hillary Clinton, and 64,285 misogynistic word usages associated with Hillary Clinton, generated between 2014 and 2018. Analyses were carried out in *R* (Version 4.1.3) using the *nlme* package. All analyses were conducted after multiplying the relative frequency of misogyny by 10^7^ due to the small numbers in this variable, to avoid the continuous use of scientific notation when reporting results. Using procedures outlined by Jebb et al.^27^ we conducted an interrupted time series analysis on the relative frequencies of misogynistic words used in tweets mentioning Clinton. Interrupted time series analyses allow researchers to examine whether time series observations are “interrupted” by incidents occurring at a known point in time, by estimating the trends of the time-series data pre- and post-event^28^. In the current study, we examined the effect of two incidents: Clinton’s announcement that she was running for president, and the 2016 election. Thus, the time series was partitioned into three periods: before Clinton announced her candidacy for president (Jan 1st, 2014–April 11th, 2015), after the announcement (i.e., the campaign period; April 12th 2015–November 8th 2016), and after the election (November 9th 2016–December 31st 2017). Our model therefore contained the predictor *time*, a mean-centered continuous variable indicating the time in days from the start of the observation period, and *period*, a factor variable that designates each time point to one of the three periods. Campaign period was chosen as the reference variable, as our hypotheses focus on comparing this period to the periods prior to and after the campaign.

To examine whether the effect of time on the relative frequency of misogyny varied across period, we included the two interaction effects of time and period into the model. These interaction effects allow us to determine whether there was a change in the *trend* of misogyny over time, after Clinton announced her candidacy and after the election. A trend of rising misogyny during the campaign period would provide support for the hypothesis that misogyny towards women is exacerbated when they challenge the gender status-quo. However, a stronger test of this hypothesis compares the slopes of misogyny across the three periods, expecting in particular that the increasing level of misogyny during the campaign period will be stronger when compared to trend of misogyny before the campaign.

Finally, to examine whether attention to Clinton was related to misogyny towards her, we included the variable *Hillary attention* as a predictor*,* which references the summed daily frequencies of the three Clinton search terms. The final model was therefore specified with the formula (Misogyny $\sim$ Time*Period + Hillary attention). We used a generalized least-squares (GLS) regression model with an AR1 component, which accounts for autocorrelation by predicting each day’s level of misogyny based on the immediately preceding value.

## Results

**Figure 2:**
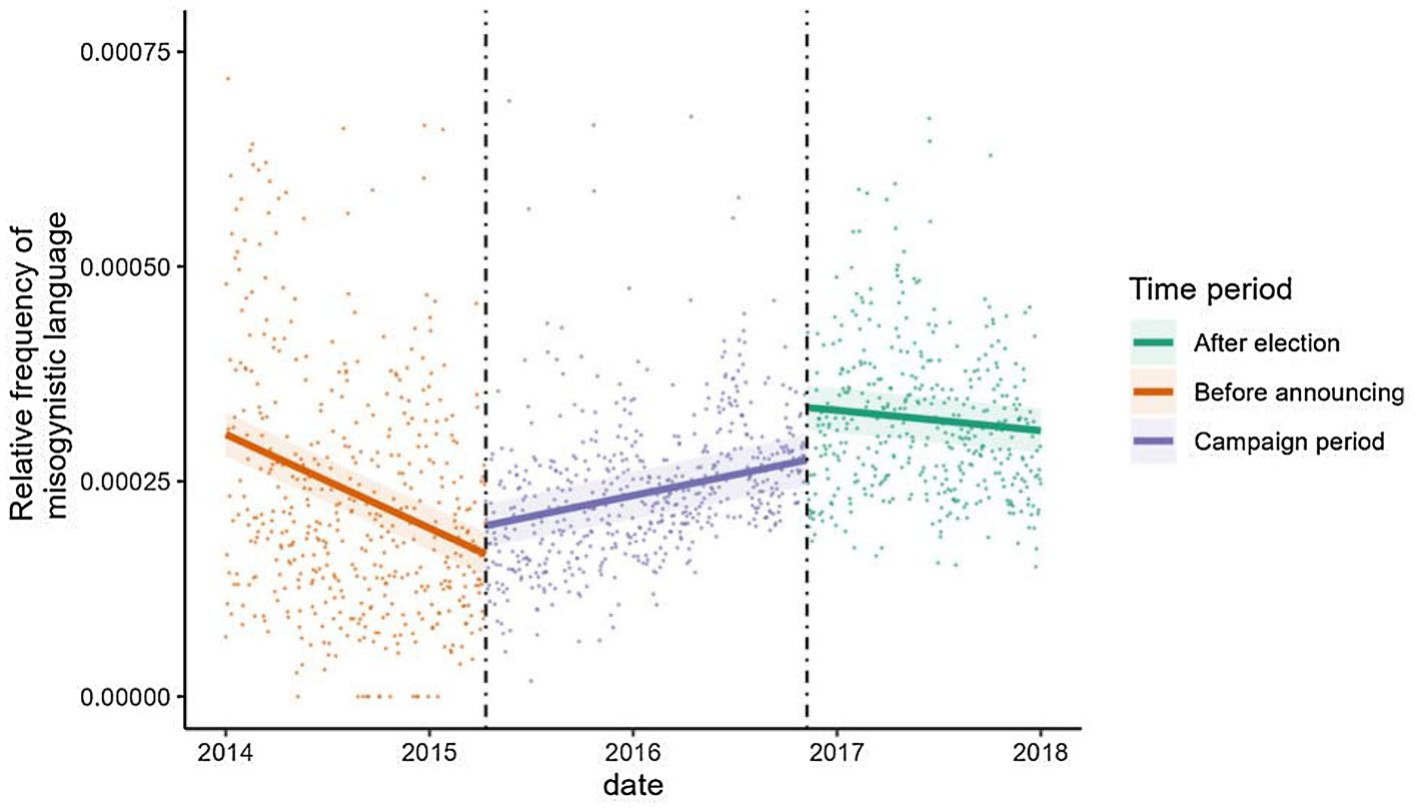
The observed and predicted relative frequency of misogynistic language among tweets about Hillary Clinton. *Note.* Shaded areas refer to 95% confident intervals.

Model results are reported in Table 1. Results indicate there was a significant, positive main effect of time on the relative frequency of misogynistic language directed towards Clinton. However, this was qualified by an interaction with period (See Fig. 2). The interaction effect comparing the slope during the campaign period to the slope before announcing found that these slopes differed significantly. Simple effects reveal that before announcing, there was a significant negative trend in the relative frequency of misogyny *b* = − 2.987, *SE* = 0.467, *t*(1453) = − 6.394, *p* < 0.001. However, during the campaign period, this trend became positive, *b* = 1.392, *SE* = 0.421, *t*(1453) = 3.306, *p* = 0.001. A comparison of the intercepts of the two slopes on the day of the campaign announcement revealed that there was no significant stepwise increase in the relative frequency of misogynistic tweets, *b* = 319.909, *SE* = 170.521, *t*(1453) = 1.876, *p* = 0.061). Taken together, these results suggest that the campaign announcement represented an inflection point in the relative frequency of misogyny towards Clinton, marking the time at which it began to rise.

**Table 1:** Regression output from interrupted time series model investigating the effect of time, period, and Hillary attention on the relative frequency of misogynistic language among tweets about Hillary Clinton.

Effect	Estimate	*SE*	*t*	*p*
Time	1.392	0.421	3.306	0.001**
Period (campaign vs. before announcing)	− 1478.165	250.875	− 5.892	< 0.001***
Period (campaign vs. after election)	1223.325	298.974	4.092	< 0.001***
Time x period interaction (campaign vs. before announcing)	− 4.379	0.627	− 6.985	< 0.001***
Time x period interaction (campaign vs. after election)	− 2.015	0.700	− 2.878	0.004**
Hillary attention	0.009	0.004	2.038	0.042*
AR1 component (Φ)	0.231			

*p < 0.05, **p < 0.01, ***p < 0.001.

The interaction effect comparing the slope during the campaign period to the slope after announcing demonstrate these slopes significantly differ. In contrast to the positive slope during the campaign period, simple slopes analyses reveal there was no association between time and the relative frequency of misogyny after the election *b* = − 0.624, *SE* = 0.548, *t*(1453) = − 1.138, *p* = 0.256. A comparison of the intercepts of the two slopes on the day of the election revealed a significant stepwise increase in the relative frequency of misogynistic tweets, *b* = 593.527, *SE* = 199.203, *t*(1453) = 2.980, *p* = 0.003). Together, these results suggest that the election was associated with a significant immediate increase in the level of misogyny towards Clinton, though the long-term effect was to stabilize the trend of misogyny over time.

As can be seen in Table 1, the model also uncovered a small but significant positive relationship between the relative frequency of Hillary mentions (Hillary attention) and the relative frequency of misogyny. However, this effect became non-significant when excluding 12 outliers from the analysis, identified using the *tsoutlier* package, which implements an iterative procedure of anomaly identification and model estimation to identify outliers in time-series data, based on the approach described in Chen and Liu^29^. All other effects remained statistically identical in the model that excluded outliers, except for the step-wise increase in Twitter misogyny on the day of the campaign announcement, which became statistically significant (*b* = 332.413, *SE* = 152.92, *t*(1453) = 2.174, *p* = 0.030; see supplemental materials) (Table S1).

## Discussion

The current study examined the relative frequency of misogynistic tweets associated with Hillary Clinton before, during, and after her 2016 presidential campaign. We found that in the period before the presidential campaign, misogyny towards Clinton decreased over time. However, this trend reversed with Clinton’s campaign announcement, after which misogyny steadily increased over time and only plateaued after the election, when the threat of Clinton’s electoral success had subsided. In tandem, these results suggest that Clinton’s attempt to gain political power initiated the online misogyny directed towards her, consistent with the notion that misogyny is a backlash response to the threat female politicians pose to male political dominance.

Our results demonstrate that Clinton’s campaign announcement represents a clear inflection point, marking the time at which misogynistic language towards Clinton started to rise. However, we did not find evidence that the announcement was associated with a significant *immediate* step change in misogynistic language, except in the analysis when outliers were excluded. The absence of this effect is arguably not congruent with one possible reading of backlash theory, which might predict an immediate exacerbation of penalties for power-seeking female politicians^11^. However, backlash theory makes no specific claim about the immediacy or time course of penalties, so the lack of a step change at the time of Clinton's announcement is not inconsistent with backlash as a gradual, crescendoing process.

The temporal nature of increasing misogyny throughout the campaign aligns with other research investigating emotional reactions to Clinton throughout the 2016 election. Miller and Borgida^30^ found that participants with a stronger gender-system justification motive became significantly more negative in their evaluations of Clinton as the election grew closer. Our research builds on this work, providing further evidence that moving closer to a gender system-threatening event (like the 2016 election) can increase the propensity for system justifying behaviors, like online misogyny.

We did not predict that Twitter misogyny would decline prior to Clinton’s campaign announcement. However, speculating through the lens of backlash theory, the decline may reflect diminishing perceptions of Clinton as a dominant public figure prior to the 2015 campaign announcement. Clinton did not hold a public position between Jan 2014 and April 2015, as she left her position as U.S. Secretary of State on February 1st, 2013. Thus, perceptions of her as a dominant, political figure likely waned in the years prior to her campaign announcement, which may have led to the decreasing Twitter misogyny observed during this period.

Surprisingly, our results show that Clinton’s election loss was associated with an immediate increase in misogyny, which subsequently extended into a longer-term stable trend. Whilst this is not predicted by backlash theory, it is plausible that the immediate increase in misogyny is an outcome of schadenfreude; the emotional pleasure derived from another’s misfortune. Prior research has found that schadenfreude is elicited by high-status, competitive targets—like dominant women—and is associated with antisocial motives for following others on social network sites, as well as online trolling^31–33^. A qualitative investigation of tweets surrounding #gamergate—an online harassment campaign that significantly negatively impacted two female gamers—also found evidence that the internet discourse frequently displayed schadenfreude and resentment^34^. To further investigate this theory, future research could experimentally test whether the misfortunes of dominant women are particularly likely to result in online misogyny via schadenfreude.

The relationship between attention and misogyny towards Clinton was weak, and once excluding outliers, non-existent. Therefore, our hypothesis that increased visibility of Clinton’s role incongruity would be associated with greater misogyny was not fully supported. It is possible, however, that Clinton’s high profile created a ceiling effect with regards to this dimension of role incongruity. To test the link between attention and misogyny more fully, future research could explore the trajectory of misogyny and social media attention among lesser-known female political candidates. It’s also possible that the association between attention and misogyny would be stronger when examining less explicit forms of misogyny, not captured in our dictionary of misogynistic terms. Future research could use a combination of manual coding and machine learning techniques to identify more subtle forms of online misogyny and examine associations with female leader visibility^35,36^.

The relative frequencies of misogyny in our study are small due to our normalization process, which examined the use of 20 misogynistic words as a proportion of all possible words generated in tweets mentioning Clinton. These proportions should be interpreted with caution, as we did not aim to comprehensively measure the prevalence of Twitter misogyny towards Clinton, but rather, aimed to examine whether the most severe, explicit forms of misogyny increased at critical junctures in time, as predicted by backlash theory. Whilst the proportions of misogynistic words at the extreme end of this continuum are small, their prevalence and rise are important to study, as they contribute to a hostile environment that can impact the willingness of female candidates to continue their work, and can legitimize hostility towards female politicians among extremists who may be contemplating violence^4,37^.

It is important to note that our research only focused on Hillary Clinton, and as such, research on the misogyny experienced by other female leaders is needed to ensure our findings generalize beyond this case study. Additionally, whilst our approach was able to measure real-world reactions to Clinton and therefore achieves high ecological validity, our analysis was observational and thus has limitations to fully reveal causal mechanisms. For example, we cannot rule out the possibility that the increasing misogyny throughout the campaign was due to an increase in general antipathy towards Clinton, or a shift in norms that increased the acceptability of Twitter misogyny towards Clinton, as opposed to backlash in response to Clinton’s gender role violation. Future research should test the relationship between female dominance and online misogyny in experimental, lab-based studies to confirm the causal processes underlying our quasi-experimental results. Another important avenue for future research concerns the impact of other, intersecting social structures and norms on online misogyny and harassment. For example, prior research suggests that women of color experience an increased risk of workplace sexual harassment^38^. As female politicians of color threaten not only the gender, but also racial status quo, future research could examine whether the frequency, and content, of misogyny differs depending on the racial identity of the female political leader.

## Conclusion

Examining online misogyny towards Hillary Clinton from 2014 to 2018, we found that the downwards temporal trajectory of misogyny reversed when Clinton announced her presidential campaign, and subsequently maintained a steady increase that only abated after the election, when Clinton’s power seeking behavior had ceased. These findings are consistent with the notion that online misogyny towards female political nominees is a form of backlash prompted by their ambition to represent the public in the political arena.

## Supplementary Information

Supplementary Information.

## Data Availability

The data that support the findings of this study are openly available in the Open Science Framework at [https://osf.io/mbkj5].

## References

[1] Beinart, P. Fear of a female president. *The Atlantic* https://www.theatlantic.com/magazine/archive/2016/10/fear-of-a-female-president/497564/ (2016).

[2] Hall, R. Hillary Clinton faced constant sexism in 2016 campaign, says ex-aide. *The Guardian* https://www.theguardian.com/us-news/2022/jun/03/hillary-clinton-faced-constant-sexism-in-2016-campaign-says-ex-aide (2022).

[3] Mikolajczak, G., Carson, A. & Ruppanner, L. Sexism, harassment, bullying: just like federal MPs, women standing for local government cop it all. *The Conversation* https://theconversation.com/sexism-harassment-bullying-just-like-federal-mps-women-standing-for-local-government-cop-it-all-157396 (2021).

[4] Carter, A. & Sneesby, J. Mistreatment of women MPs revealed. *Br. Broadcast. Corp. News* https://www.bbc.com/news/uk-politics-38736729 (2017).

[5] Vijeyarasa R (2022). The Woman President: Leadership, Law and Legacy for Women Based on Experiences from South and Southeast Asia.

[6] Rudman LA, Moss-Racusin CA, Glick P, Phelan JE, Devine P, Plant A (2012). Reactions to vanguards: Advances in backlash theory. Advances in Experimental Social Psychology.

[7] Mishra S, Kray LJ (2022). The mitigating effect of desiring status on social backlash against ambitious women. J. Exp. Soc. Psychol..

[8] Rudman LA, Moss-Racusin CA, Phelan JE, Nauts S (2012). Status incongruity and backlash effects: Defending the gender hierarchy motivates prejudice against female leaders. J. Exp. Soc. Psychol..

[9] Prentice DA, Carranza E (2002). What women and men should be, shouldn’t be, are allowed to be, and don’t have to be: The contents of prescriptive gender stereotypes. Psychol. Women Q..

[10] Koenig AM (2018). Comparing prescriptive and descriptive gender stereotypes about children, adults, and the elderly. Front. Psychol..

[11] Okimoto TG, Brescoll VL (2010). The price of power: Power seeking and backlash against female politicians. Personal. Soc. Psychol. Bull..

[12] Yildirim TM, Kocapınar G, Ecevit YA (2021). Status incongruity and backlash against female legislators: How legislative speechmaking benefits men, but harms women. Polit. Res. Q..

[13] Bauer NM, Harman M, Russell EB (2022). Do voters punish ambitious women? Tracking a gendered backlash toward the 2020 democratic presidential contenders. Polit. Behav..

[14] Manne K (2017). Down Girl: The Logic of Misogyny.

[15] Berdahl JL (2007). The sexual harassment of uppity women. J. Appl. Psychol..

[16] Mantilla K (2015). Gendertrolling: How Misogyny Went Viral.

[17] Pew Research Centre. Americans who mainly get their news on social media are less engaged, less knowledgeable. https://www.pewresearch.org/journalism/2020/07/30/americans-who-mainly-get-their-news-on-social-media-are-less-engaged-less-knowledgeable/ (2020).

[18] Blake KR, O’Dean SM, Lian J, Denson TF (2021). Misogynistic tweets correlate with violence against women. Psychol. Sci..

[19] Fudolig MI, Alshaabi T, Arnold MV, Danforth CM, Dodds PS (2022). Sentiment and structure in word co-occurrence networks on Twitter. Appl. Netw. Sci..

[20] Rathje S, Van Bavel JJ, van der Linden S (2021). Out-group animosity drives engagement on social media. Proc. Natl. Acad. Sci. U.S.A..

[21] Williams MJ, Tiedens LZ (2016). The subtle suspension of backlash: A meta-analysis of penalties for women’s implicit and explicit dominance behavior. Psychol. Bull..

[22] Rheault L, Rayment E, Musulan A (2019). Politicians in the line of fire: Incivility and the treatment of women on social media. Res. Politics.

[23] Dodds P (2022). Fame and Ultrafame: Measuring and comparing daily levels of ‘being talked about’ for United States’ presidents, their rivals, God, countries, and K-pop. JQD.

[24] Dodds PS (2021). Computational timeline reconstruction of the stories surrounding Trump: Story turbulence, narrative control, and collective chronopathy. PLoS ONE.

[25] Rothe DL, Collins VE (2019). Turning back the clock? Violence against women and the Trump administration. Vict. Offenders.

[26] Alshaabi T (2021). Storywrangler: A massive exploratorium for sociolinguistic, cultural, socioeconomic, and political timelines using Twitter. Sci. Adv..

[27] Jebb AT, Tay L, Wang W, Huang Q, Croudace TJ (2015). Time series analysis for psychological research: Examining and forecasting change. Front. Psychol..

[28] Cook TD, Campbell DT (1979). Quasi-Experimentation: Design and Analysis Issues for Field Settings.

[29] Chen C, Liu L-M (1993). Joint estimation of model parameters and outlier effects in time series. J. Am. Stat. Assoc..

[30] Miller AL, Borgida E (2019). The temporal dimension of system justification: Gender ideology over the course of the 2016 election. Personal. Soc. Psychol. Bull..

[31] Brubaker PJ, Montez D, Church SH (2021). The power of schadenfreude: Predicting behaviors and perceptions of trolling among Reddit users. Soc. Med. Soc..

[32] Cikara M, Fiske ST (2013). Their pain, our pleasure: Stereotype content and schadenfreude: Stereotype content and schadenfreude. Ann. N. Y. Acad. Sci..

[33] Ouwerkerk JW, Johnson BK (2016). Motives for online friending and following: The dark side of social network site connections. Soc. Med. Soc..

[34] Mortensen, T. E. & Sihvonen, T. Negative emotions set in motion: The continued relevance of #gamerGate. in *The Palgrave Handbook of International Cybercrime and Cyberdeviance* 1–23 (Springer International Publishing, 2020). 10.1007/978-3-319-90307-1_75-1

[35] Amnesty International. *Troll Patrol Findings.* https://decoders.amnesty.org/projects/troll-patrol/findings (2019).

[36] Delisle, L. *et al.* A large-scale crowdsourced analysis of abuse against women journalists and politicians on Twitter. Preprint at http://arxiv.org/abs/1902.03093 (2019).

[37] Krook ML, Sanín JR (2020). The cost of doing politics? Analyzing violence and harassment against female politicians. Perspect. Polit..

[38] Cassino D, Besen-Cassino Y (2019). Race, threat and workplace sexual harassment: The dynamics of harassment in the United States, 1997–2016. Gend. Work. Organ..

