# Supplementary material for: Twitter misogyny associated with Hillary Clinton increased throughout the 2016 U.S. election campaign
Source: Sci Rep. 2023 Mar 31;13:5266. doi: 10.1038/s41598-023-31620-w (PMC10066361; doi:10.1038/s41598-023-31620-w)
Supplement: Supplementary file 1 — Supplementary Information. [file 41598_2023_31620_MOESM1_ESM.docx]

**Supplemental Materials**

**Misogynistic Terms**

- Battleaxe
- Bimbo
- Bitch
- Cuck
- Cunt
- Ho
- Hobag
- Hoe
- Hussy
- Hussi
- Nympho
- Prude
- Psycho
- Skank
- Slag
- Slut
- Tramp
- Twat
- Whore
- Witch

**Outlier Analyses**

Outliers were identified among the time series of relative frequencies of misogynistic language using the *tsoutliers* package, which implements an iterative procedure of anomaly identification and model estimation based on the approach described in Chen and Liu^[1]^. This method identified 12 outliers described in the table below. Most of the days did not correspond with any definitively notable events, apart from the 22^nd^ of October 2015, when Clinton testified at an all-day Benghazi hearing, the 14^th^ of April 2016, when Clinton debated Bernie Sanders for the Democratic presidential nomination, and on the 2^nd^ of October 2017, when Hillary Clinton criticized existing gun laws on Twitter.

**Supplementary Table S(1).**

*Identified Outliers*

| outlier | date |
| --- | --- |
| 5 | 5/01/2014 |
| 26 | 26/01/2014 |
| 212 | 31/07/2014 |
| 264 | 21/09/2014 |
| 356 | 22/12/2014 |
| 357 | 23/12/2014 |
| 390 | 25/01/2015 |
| 509 | 24/05/2015 |
| 660 | 22/10/2015 |
| 835 | 14/04/2016 |
| 1234 | 18/05/2017 |
| 1371 | 2/10/2017 |

We re-ran the time-series analyses after replacing the outlier values with model estimates^[1]^. Results largely remained the same as the initial analyses, indicating there was a significant, positive main effect of time on the relative frequency of misogynistic language directed towards Clinton. However, this was qualified by an interaction with period. The interaction effect comparing the slope of time during the campaign period to the slope of time before announcing found that these slopes significantly differed from each other. Simple effects revealed that before announcing, there was a significant negative trend of time on the relative frequency of misogyny *b* = -2.950, *SE* = 0.419, *t*(1453) = -7.042, *p* <.001. However, during the campaign period, this trend became positive, *b* = 1.556, *SE* = 0.378, *t*(1453) = 4.123, *p* <.001. A comparison of the intercepts of the two slopes on the day of the campaign announcement revealed that there was a significant stepwise increase in the misogynistic tweets, *b* = 332.413, *SE* = 152.92, *t*(1453)=2.174, *p* = .030).

The interaction effect comparing the slope of time during the campaign period to the slope of time after announcing demonstrate these slopes significantly differ from each other. As opposed to the positive slope of time during the campaign period, simple slopes analyses revealed there was no association between time and the relative frequency of misogyny after the election *b* = -0.709, *SE* = 0.492, *t*(1453) = -1.443, *p* = .149. A comparison of the intercepts of the two slopes on the day of the election revealed a significant stepwise increase in the relative frequency of misogynistic tweets, *b* = 537.841, *SE* = 178.666, *t*(1453) = 3.010, *p* = .003). Together, these results suggest that the election was associated with a significant immediate increase in the level of misogyny towards Clinton, though the long-term effect was to stabilise the trend of misogyny over time.

As can be seen in Table 2., the model also uncovered a small and only marginally positive relationship between the relative frequency of Hillary mentions (Hillary attention) and the relatively frequency of misogyny.

**Supplementary Table S(2).**

*Regression output from interrupted time series model after replacing outliers*

| Effect | Estimate | *SE* | *t* | *p* |
| --- | --- | --- | --- | --- |
| Time | 1.556 | 0.378 | 4.123 | <.001*** |
| Period (campaign vs. before announcing) | -1524.493 | 224.978 | -6.776 | <.001*** |
| Period (campaign vs. after election) | 1245.933 | 268.109 | 4.647 | <.001*** |
| Time x period interaction (campaign vs. before announcing) | -4.507 | 0.562 | -8.016 | <.001*** |
| Time x period interaction (campaign vs. after election) | -2.266 | 0.628 | -3.609 | <.001*** |
| Hillary Attention | 0.007 | 0.004 | 1.813 | .070^†^ |
| AR1 component (Φ) | .23 |  |  |  |

*Note*. ^†^*p* < .10, ****p* <.001

**References**

1. Chen, C. & Liu, L.-M. Joint estimation of model parameters and outlier effects in time series. *Journal of the American Statistical Association* **88**, 284–297 (1993).
